# What are the experiences and support needs of district nurses caring for terminally ill people with delirium at home? A qualitative study

**DOI:** 10.1186/s12904-024-01627-9

**Published:** 2025-03-08

**Authors:** Elizabeth Arnold, Jean Lugton, Juliet Spiller, Anne Finucane

**Affiliations:** 1 Marie Curie Hospice Edinburgh, 45 Frogston Road West, Edinburgh, EH10 7DR UK; 2 Clinical Psychology, School of Health in Social Science, University of Edinburgh, Edinburgh, EH8 9AG UK

**Keywords:** Delirium, Delirious, Confusion, Palliative, Terminal, District nurses, Home, Community, Prevention, Detection, Management

## Abstract

**Background:**

Delirium is a serious neuropsychiatric syndrome, which is common amongst terminally ill people in the community. District nurses have a key role in supporting terminally ill people to remain at home.

**Objectives:**

To explore the experience and support needs of district nurses caring for people with delirium in home settings.

**Methods:**

Semi-structured individual and small group interviews were conducted via Microsoft Teams with 12 district nurses in Scotland, UK. Data was analyzed using framework analysis. Data was coded both deductively and inductively.

**Results:**

Overarching themes were (i) challenges of delirium detection in the community, (ii) challenges managing delirium in the community, (iii) family carers as providers and recipients of care and (iv) education, training and support needs. Participants valued clinical judgement alone in detecting delirium, over use of formal assessment tools. Patients were referred to district nursing services at an advanced stage of their illness, with nurses needing to make rapid decisions about their care, sometimes with limited information. Participants were familiar with non-pharmacological strategies and the importance of family carer support, but uncertainty remained regarding pharmacological management of distressing symptoms. The term ‘delirium’ was rarely used. Challenges accessing timely advice and practical support from other health and social care professionals were reported. Participants identified delirium detection and the pharmacological management of persistent delirium as priorities for training.

**Conclusion:**

Caring for terminally ill people with delirium in the community is challenging. Educational interventions may be beneficial in developing district nurses’ confidence in supporting terminally ill patients and their families. Responsive advice and support are required from specialist palliative care services.

**Supplementary Information:**

The online version contains supplementary material available at 10.1186/s12904-024-01627-9.

**What is already known about this subject**.

- Delirium is a serious neuropsychiatric condition, which is common amongst terminally ill people, especially towards the end of life.
- District nurses are key in supporting terminally ill people to remain at home at the end of life.

**What this study adds**.

- As far as we are aware, this is the first study to explore the experiences of district nurses in supporting people with an advanced progressive or terminal illness, affected by delirium, in the community.
- District nurses value clinical judgement alone in detecting delirium, over use of formal delirium detection tools.
- Patients are often referred to district nursing services at a more advanced stage of their illness, than in the past, with nurses needing to make rapid decisions about their care, sometimes with limited information.
- District nurses report that hyperactive and mixed delirium are the most commonly experienced delirium subtypes, amongst their patients. 
- District nurses are familiar with non-pharmacological strategies for managing delirium, but further education and training may be beneficial regarding pharmacological management of distressing delirium symptoms and terminal agitation.

**How might this study affect research**,** practice**,** and policy**.

- District nurses identified training needs regarding delirium identification, determining the underlying causes, and management of distressing hyperactive symptoms towards the end of life.
- Improved access to specialist palliative care and other services is required to support district nurses caring for terminally ill people experiencing delirium.
- Most delirium research on terminally ill populations has taken place in inpatient settings. More research focusing on assessment and management of delirium amongst community-based terminally ill people is required.

## Background

Delirium is a serious neuropsychiatric condition [1], which is common amongst terminally ill people. Studies of community patients on their first review by specialist palliative care services suggest a point prevalence of 4–12%, rising to 42–44% towards the end of life [2]. Delirium has been described as a ‘complex medical emergency.’ [3] Its presence indicates a poor prognosis, with only half of cases being reversible in patients with advanced cancer [4]. Delirium is associated with an increased risk of hospital admission, morbidity, and mortality [5, 6], as well as causing significant distress to both patients and their families [7]. 

Palliative and generic delirium guidance exists [8–12], however recent surveys of UK and Ireland-based palliative care specialists have demonstrated that clinical practice is variable [13, 14]. For instance, there is significant variation in practice regarding delirium screening and diagnosis in UK hospices, with over one-third of physicians never using delirium clinical guidelines [14]. This may be due to lack of awareness about clinical guidelines or research, or uncertainty about their relevance to terminally ill populations. Most guidance recommends delirium screening and assessment tool use in high-risk populations [8–12], as timely detection may lead to improvements in patient outcomes, although the best tool for palliative populations remains unclear [15]. Investigation and treatment of the underlying causes of delirium are recommended, depending on the individual’s preferences for care and the stage of their disease - yet decisions concerning potential reversibility of delirium may be complex [16]. The evidence for non-pharmacological strategies is largely extrapolated from non-palliative settings, but recommended in both delirium management and prevention [16]. Pharmacological treatment is reserved to palliate distressing symptoms, although controversy and uncertainty remain, especially regarding the use of antipsychotics [3, 16–18]. 

In the UK, increasing numbers of people receive end of life care in community settings, with some projections predicting that most people will die at home or in care homes by 2040 [19–21]. Similar trends are evident in America, Canada, and Sweden [22–24]. In many countries, a shift towards dying in community settings has been accelerated by the Covid pandemic, with reductions in the proportion of hospital deaths, and increases in deaths in other settings since April 2021 [25, 26]. 

In the UK, district nursing services, as part of the primary care team, play a vital role in supporting people with palliative care needs to remain at home [27, 28]. District nurses are senior nurses, who have completed additional training to become specialist community practitioners [29]. They lead teams of community nurses and support workers to provide nursing services to housebound patients in the community, including out-of-hours (overnight and during the weekends).

District nursing services work in partnership with specialist palliative care services, where they exist. These specialist palliative care services have an important role in supporting those with more complex symptoms and psychological needs. Specialist palliative care nurses have reported challenges associated with delirium management in the community, including time limitations with patients, reliance on families and difficulties accessing medication [30]. However, to our knowledge, there are no studies exploring the perspective of district nurses, who provide much of this care, especially out-of-hours.

A qualitative systematic review and thematic analysis of the experience of delirium in palliative care settings identified 16 studies, however of these, 13 were based in hospital or hospice inpatient settings [31]. Nurses had a limited understanding of delirium, and often did not use the term ‘delirium,’ sometimes leading them to attribute delirium symptoms to other factors, such as age or personality. Of the three studies that included staff from community settings [32–34], specific themes identified were challenges to person centred care, the vital role of the family, the distressing impact of delirium on the patient and family, and challenges with decision-making regarding medication. An earlier systematic review also highlighted the key role of the family in supporting the person with delirium [35]. 

Given the considerable number of people dying in community settings [26], and the role of district nurses in providing care for people approaching end of life, we sought to explore how district nurses support patients with delirium, and whether they have training and support needs.

## Methods

### Design

Qualitative study, drawing on the epistemology of pragmatism. Pragmatism is concerned less with truth and reality, accepting that there can be single or multiple realities, and instead focuses on what works or what is useful [36]. In contrast with theoretically driven research paradigms, pragmatic qualitative studies prioritize the need to understand applications, evaluate what works, and find solutions to real-world problems [37]. This approach allowed the research team to focus on understanding district nurses’ views about how delirium is managed in the community, and what, if anything, might be needed to support this.

### Participants

Participants were district nurses and registered community staff nurses, working within district nursing services, in Scotland, UK. The term ‘district nurse’ will be used in this paper, to refer to both district nurses and community staff nurses. District nurses were eligible to participate if they had been working within district nursing services for at least 6 months.

### Setting and sampling approach

We sought a convenience sample of district nurse participants, to include those working both in- and out-of-hours, and from urban and rural settings. Participants were recruited from district nurse hubs and unscheduled care services in 10 health boards in Scotland, UK. As is often the case, the size of the sample was determined by pragmatic considerations [38], namely the availability of participants and resources to complete the study in a timely fashion.

### Recruitment

Nursing directors of twelve health boards in Scotland, UK, were contacted about the study. The nursing leads of ten health boards disseminated information by email, through their district nursing networks. The ‘invitation to participate’ email included the participant information sheet, and a link to the online consent form and participant characteristics questionnaire. The study was also publicized at district nurse educational events and community palliative care meetings,  in the same health board as the lead researcher (EA). The district nurse collaborator (MJR) also assisted with promotion of the study, in the same health board. The online consent form was completed prior to nurse participation.

### Data collection

We conducted individual or small group semi-structured interviews using Microsoft TEAMS, between September 2021 and April 2022. Questions explored the participants’ understanding of the term ‘delirium’, delirium prevention strategies, assessment of delirium, including awareness and use of screening tools, and their experience of delirium management. (Supplementary file 1, Table 1 - Draft interview schedule.) The interview schedule was developed by the research team, which included two clinicians (EA, JS), and an experienced district nurse (MJR). The small group interviews were conducted by the lead researcher (EA). EA is a palliative care doctor with 15 years’ experience, including working in the community with district nurses. EA also has a research role and has conducted research on delirium for over 5 years. EA was supervised by AF, an experienced researcher, throughout the process. Only one participant was known to the interviewer in advance of the study. Interviews were transcribed, with any potentially identifiable data removed during this process.

### Data analysis

Data was analysed using a framework approach [39] in NVivo 14. This is a codebook approach, which involves applying codes and categories to data within themes. Themes may be inductively or deductively developed. Four transcripts were read and coded independently by two members of the research team (EA and AF). Each researcher read the whole transcript, making notes as appropriate. After familiarization, each researcher read the transcript line by line, applying a code, describing what they had interpreted in the text as being relevant or important. Coding was both inductive and deductive. As we were particularly interested in findings pertinent to delirium assessment, management, and training needs, these broad categories were pre-defined, and text was indexed in relation to these categories. However, to ensure important aspects of the data were not missed, we also allowed for open coding, and the inclusion of codes that had not been pre-defined. After independent coding, researchers (EA and AF) met to compare the codes that had been applied, and to agree on a set of codes to apply to all transcripts. Codes were then grouped into categories to form the working analytical framework. This framework was then applied to all transcripts by EA. There was scope for further coding throughout the process, and coding was not limited to the initial framework. Framework matrices were used to summarise data by category and code across interviews. Trustworthiness was enhanced by the involvement of another two members of the research team in data analysis (JS and JL) [40]. 

### Patient and public involvement

Patient and public representatives were engaged in the study’s planning, and included relatives of terminally ill people, one of whom had experienced delirium prior to death. Public representatives were members of the Marie Curie Research Voices group, a group established to provide feedback on Marie Curie research projects. Patient and Public Involvement (PPI) representatives considered delirium management in the community a research priority, and provided feedback on the proposed methodology.

## Results

### Participant characteristics

Sixteen district nurses were recruited, however 4 withdrew prior to the interviews, due to workload demands. Twelve participants completed an individual or small group interview (Supplementary file 1, Table 2 - Participant characteristics). They comprised 9 district nurses and 3 community nurses. Five of the 12 participants were recruited from the same health board as the researcher EA, with the remaining 7 participants based in 6 other Scottish health boards. Most were experienced district nurses, with at least 8 years experience in their role, and a quarter (3/12) were non-medical prescribers. Most worked during the daytime (typically 8:30am-4:30pm) and early evening (4:30 − 8:30pm), with 2 participants working for the overnight and weekend unscheduled care services.

We present findings in relation to four main themes. These are: challenges in delirium detection, uncertainties around delirium management, family-focused care, and education and training needs. (Supplementary file 1, Table 3 – Themes, categories and codes.)

### Theme 1: Challenges of delirium detection in the community

#### Delirium detection tools not commonly used

Participants relied on their clinical judgement, when determining whether the person had delirium. Their assessment was based on how the patient presented, as well as information from family carers and other professionals.

*‘I rely on the carers telling me (about any) change in (the patient’s) condition*,* (and) the patient themselves*,* whether there’s any confusion … so we’d be looking at that whole picture of the patient… What are the triggers? What’s different from yesterday or what’s different from last week?’ P4*

Most participants reported being aware of delirium detection tools, most commonly the 4AT [41], but remained unconvinced about their value. Their opinion was that, when delirium occurs, the diagnosis is often clear, thus a tool adds little value beyond clinical judgement. Participants also reported a formal detection tool might be inappropriate when patients are unable to communicate.

*‘I don’t routinely use them. But I would say normally …. when we go to see people and there is an issue with delirium… if you were taking a 4AT*,* they would be a ‘4’ (score of 4 indicates possible delirium)*,* because they’re not really able to answer the questions very much.’ P1*

This was viewed as particularly the case for patients towards the end of life, when experiencing reduced levels of consciousness. The patient’s delirium diagnosis was deemed obvious or considered irreversible.

*‘…it’s more the norm now that we’re getting patients right at the end of life…and then the 4AT becomes slightly irrelevant*,* because it’s too late.’ P7*

*‘We don’t really use a tool as such*,* because it’s so end-stage*,* that any of the tools*,*… like…(the) 4AT…*,* they’re not really appropriate at that stage*,* because we are talking (about the) last couple days of life usually*,* so it is clinical judgement.’ P2*

#### Lack of confidence using delirium terminology

Participants reported a tendency to document delirium-related symptoms, without labelling as ‘delirium.’ Most reported they recognised the symptoms, but sometimes lacked the confidence to use the term ‘delirium.’ For some, this was due to their perceived lack of expertise regarding delirium assessment and recognition, or the view that diagnosis was the responsibility of other healthcare professionals. This could lead to delirium being under-recognised.

*‘In district nursing*,* I think it is not something that people have expertise in generally. I think people are just dealing with symptoms as they come.’ P6.*

*‘If I’m honest*,* I think it (delirium) is not something that’s diagnosed. I don’t think we identify it as well as we should. I think …(nurses)…will more speak about people being morphine naïve, or it’s the side effects of the cancer, or the side effects of the drugs or …their condition*,* rather than thinking it’s delirium’. P4*

Reluctance to use the term ‘delirium’ was sometimes related to concerns about mistakenly identifying someone as having, or not having delirium. Ambiguity with delirium terminology was reported by some, with alternative terms such as ‘terminal agitation’ or ‘agitation’ more commonly used towards the end of life.

*‘You know you can put ‘I think this is delirium’ in the notes…I think…nurses are always frightened of getting pulled up for something*,* they’re always frightened of being wrong or somebody coming in and second-guessing them…I think unfortunately ‘delirium’ is not a word that tends to get used an awful lot… It tends to be ‘terminal agitation’*,* ‘uncontrolled symptoms.’ P2*

However, use of the term ‘delirium’ in discussions between healthcare professionals was perceived as potentially improving support for both patients and their families.

*‘Everything doesn’t have to have a name*,* but it makes it a little bit easier if you can…say*,* ‘Right*,* I think that they have got delirium*,* therefore this is what we have to do.’ So*,* I suppose recognising that it is that by whatever means…and then how do we then make it better*,* for not just the patients*,* for the relatives as well.’ P9*

### Theme 2: Challenges managing delirium in the community

#### Limited information

Participants reported terminally ill people were increasingly referred to their services with more advanced disease, which led to less time and fewer opportunities to develop relationships with patients and their families. Participants reported having less knowledge about the person, their family, and their preferences,  which sometimes made decisions about their care more challenging.

*‘In an ideal world*,* we’d start that connection really early in their illness*,* earlier in their journey*,* and get to know them and their families and their wishes - but lately*,* what we’ve been finding is we’re just meeting them nearer the terminal phase*,* so we don’t know them and their families … or what their wishes are.’ P8*

This was particularly the case for those working out-of-hours. District nurses, with the out-of-hours services, described limited access to patients’ electronic medical records, either because the information was out-of-date, or mobile devices for remote access were unavailable.

*‘There is quite a difference between caring for people during the day and at night*,* in that*,* at night*,* it’s…quite frequent where(by) you don’t know who you’re going into (visit). Sometimes (you’re) going in with very little information…You’re going into people who are very distressed*,* whose family are very distressed*,* and they want something done quickly*,* and there’s a certain amount of ‘right*,*…what’s actually going on here.’ P1*

#### Complex symptom management as disease progresses

Participants recognised the wide range of causes of delirium in terminally ill patients. Urinary retention, constipation, infection, uncontrolled pain, and medication were identified as commonly explored, potentially treatable causes. Nurses reported management was dependent on the patient’s stage of their illness and disease trajectory. Treatment of potentially reversible causes of delirium was more likely during the early phase of their terminal illness, whereas management of distressing symptoms became more important towards the end of life.

*‘It’s difficult…because it depends on what stage this person is at. Are we in the final days of life or is it something that we think we’re going to be able to reverse? … So*,* I suppose it’s always about that very person-centred care*,* looking at your patient*,*…seeing…where they are exactly at that point*,* what we think is the cause*,* can we reverse it*,* (or) are we looking at making them comfortable. What is the kindest thing for that person and their family.’ P3*

Most participants gave examples of treating distressing delirium symptoms or agitation. Hypoactive delirium was only mentioned by one participant. This may have been due to the frequency of visits to support patients experiencing hyperactive or mixed delirium.

‘*What we get called out for is probably mixed or hyperactive delirium, that is unmanageable.’ P2*

Complex, persistent delirium symptoms were commonly discussed and deemed particularly distressing for families.

*‘We see people that quite frequently (are)… very agitated*,* and that’s again why families call us*,* because they can’t cope with that.’ P1*

Participants reported challenges managing persistent distressing delirium symptoms, especially towards the end of life, when they felt unable to provide the level of care and support, they wanted to.

*‘I think the worst challenge for me is when we can’t get on top of it. When…no matter what we do*,* and no matter who we bounce back and forth with to get advice*,* we don’t get on top of it and give them that good (death) that we always try to give people.’ P7*

*‘When the families are really upset*,* I think that’s really difficult*,* because all you want for them is what they want*,* which is their relative to be comfy (comfortable)*,* settled*,* and sometimes they’re not.’ P8*

Pharmacological management, using benzodiazepines and/or antipsychotics, was advocated for delirium associated with severe distress, if there had been a poor response to non-pharmacological strategies, particularly towards the end of life, or due to concerns about patient safety.

(Regarding decision-making about use of pharmacological management)

*‘Looking at …the patient in front of you and assessing their needs and dealing with them… if they are becoming agitated…if they are becoming unsafe*,* lashing out*,* going to hurt themselves*,* things like that. Hospital beds are great*,* but if… they’ve got cot sides and they want to climb over them and are bashing their arms…so*,* …you get to a stage when you’re thinking about the patient’s safety*,* as well as their comfort*,* and the family’s distress.’ P3*

Regarding use of anticipatory medication towards the end of life, midazolam was the first-line treatment for relieving distress and ‘agitation,’ although it was unclear if this agitation was associated with delirium, or other distressing symptoms. The antipsychotic levomepromazine was recognised as a second-line medication for managing distressing delirium symptoms by a minority of participants, but typically on advice from palliative care specialists, when there was a high risk of delirium developing, or delirium was already present.

*‘We had a lady the other day…and…they (the specialist palliative care service) had suggested an agitation dose of levomepromazine*,* because she was at risk of delirium at the end of her life. So*,* we had midazolam and levomepromazine in her toolkit*,* for if this person needed them.’ P8*

Challenges of managing pain and other symptoms, alongside delirium, were described, particularly towards the end of life. Specifically, nurses described uncertainty about managing delirium associated with opioid toxicity. Advice from other healthcare professionals was considered beneficial at times.

*‘We talk about opioid use making it (delirium) worse*,* but then you need to give opioids to control the pain. They talk about steroids making it worse*,* but then we are using steroids… We are talking about making sure someone passes urine*,* but don’t put a catheter in - so you know there is a lot of conflicting information out there*,* so I think any input (from other healthcare professionals) would be good.’ P2*

#### Use of non-pharmacological strategies

District nurses were familiar with non-pharmacological strategies in supporting patients with delirium. Most participants discussed the importance of a calm environment. Providing reassurance to the terminally ill person and their family was a key strategy in alleviating fear and anxiety. Managing noise levels and limiting visitors were also discussed.

*‘I think having a calm atmosphere at home and just responding to the patient in a really calm manner*,* not having too many people in the room at the same time*,* and keep things familiar*,* you know*,* not changing anything around that would upset them*,* and at times*,*…play music in the background*,* (but) if sometimes they’re irritated by noise*,* then (do) not have that. So*,* it is really about assessing what the patient needs to help them to be calm.’ P6*

*‘Keeping calm*,* reassuring them*,* making sure the room is a nice ambient temperature*,* putting some calming music on*,* if you know something that they may be like*,* and they are familiar with.’ P7*

Participants also mentioned ensuring the person wore their glasses or hearing aids, if they needed them, as well as encouraging a good sleep routine.

*‘Ensuring they’ve got their hearing aids in*,* their glasses on; trying to alleviate fear…Calm environment - I would try to adjust the lighting for day and night.’ P2*

### Theme 3: Role of family carers as both provider and recipient of support

#### The family as care providers

Given district nurses’ limited time on home visits, and the fluctuating nature of delirium symptoms, families were recognized as vital in supporting care at home. There was a reliance on family carers, to provide collateral history when assessing patients.

*‘We would be asking the family*,* plus the patient  ‘what is the difference you see?’…Is it (a) change in their behaviour*,* or eating*,* drinking habits*,* or (are they) up during the night… So*,* looking at all these aspects of why they’ve changed.’ P4*

District nurses sometimes discussed non-pharmacological strategies with family carers. ‘Safety netting’ was also important, such that families knew when, and how to seek further support, which was typically if distressing delirium symptoms persisted or recurred.

*‘Ensuring they’ve got the correct [telephone] numbers…being clear about how many times they should give the medication before they seek advice from ourselves or the out-of-hours team*,* and just ensuring that they feel supported with whatever is going on with that situation.’ P3*

A small number of participants reported occasions when families had asked them to administer medication to patients, when they considered this inappropriate. These requests were typically prompted by family carer distress. Participants were clear they should only administer medication if clinically indicated, and further assessment of the patient, explanation, and reassurance to family carers was usually helpful in these situations.

*‘I can only treat what I assess as appropriate. Most of the time*,* patients and families*,* they understand.‘ P8*

*‘We always say… if we walk out the door and it starts again*,* we will come back you know. It is a tough one though.’ P7*

#### The family carer as recipient of support

Participants reported that when a patient had delirium, it was often distressing, tiring and stressful for the family.

*‘Generally*,* you enter a house where there is all chaos erupting and people are very upset.’P1 ‘It can be tiring for the family*,* you know*,* if somebody is trying to…come out of bed or begins to wander.’ P4*

*They (family) just want their relative to be really settled and comfy (comfortable)…Clawing at the bedsheets*,* trying to climb out of the bed*,* stuff like that*,* it (gets) carers…really*,* really upset.’ P8*

Participants described giving advice and explanation to family carers about the patient’s delirium diagnosis. There was recognition that these experiences could have negative sequalae, even after the person’s death.

*‘I suppose it’s just a lot about supporting him (as the family carer) and trying to reassure him*,* what is going on*,* what’s …causing this …*,* why she is behaving in that way. Those things can affect his grieving process and how is he going to cope after the death of his wife.’ P3*

### Theme 4: Education, training and support

#### Support from health and social care services

District nurses described the functional status of patients deteriorating with the onset of delirium, and carers struggling to support their care, leading to an urgent need for additional community support, such as packages of care. When these were unavailable, it added to the existing pressures on family carers, potentially risking the patient being able to remain at home.

*‘I suppose one of the big problems just now is the care. Can we get the care for that person? Because it could be that the family have been managing…then they develop a delirium and it affects…their mobility*,* their continence*,* … and then the family struggle. So I suppose care impacts that decision (regarding place of care) and also perhaps the family feeling that they get to a stage where actually they can’t manage*,* and I think like I’ve said previously*,* having the opportunity to spend quality time with your loved one at the end of life*,* as opposed to feeling very stressed and not managing to get that quality time.’ P3*

Timely partnership working with general practitioners (GPs) and specialist palliative care services (including advisory, home visits and overnight nursing services) were valued by district nurses, when caring for terminally ill people with complex delirium symptoms.

*‘I think it’s prompt action*,* prompt intervention*,* prompt reviewing as well by the GPs (and) the palliative care people (specialists)*,* … getting carers in quickly as well*,*… all these things help to make the family feel more supported.’ P2*

However, shortfalls in these services contributed to the challenges experienced by district nurses. Specialist palliative care services were described as limited or unavailable, and GPs were, at times, unable to visit patients at home. Reduced in-person reviews by other healthcare professionals were perceived as problematic for district nurses, especially when needing a second opinion.

*‘There are times when we just want someone else to come out and assess our patient in person*,* because we’ve done all we can*,* we’ve re-iterated all we can back to the … (GP or specialist palliative care service). Please just come and put your eyes on them and see what you think.’ P7*

#### Education and training priorities

Priorities for learning focussed on delirium identification, given the perception of under-recognition. Investigation and management of reversible causes of delirium and pharmacological strategies were also considered priorities.

*‘I don’t think people always address the delirium*,* they…maybe see (it) as a different symptom…In district nursing*,* I think it is not something that people have expertise in generally. I think people are just dealing with symptoms as they come.’ P6*

*‘Maybe workshops for our staff in recognising*,* managing*,* and knowing what reversible causes are*,* because I feel like when somebody is in that way (with agitated delirium)*,* everybody just … (administers) midazolam…There could be lots of other things we could do.’ P8*

Regarding management of distressing, hyperactive delirium symptoms,  district nurses wanted clarity about the course of action, if first-line medication was ineffective.

*‘I…find it quite frustrating that sometimes people*,* like recently a lady…she had had…four out-of-hours calls overnight*,* and … (nurses) kept giving her… midazolam*,* and clearly she wasn’t responding to that.’ P3*

#### Education and training delivery format

Some district nurses described interactive, small group sessions, delivered remotely, as the preferred format, partly because online sessions were easier to join, given their workload demands.

*‘I think an online (Microsoft) Teams thing is always better…potentially being able to ask questions*,*…having groups of district nurses there together*,* …(able to) feed off each other*,* one would say something, and you’d (say) ‘gosh yeah*,* that’s a good point*,* I never thought about that.’…A leaflet is fine*,* but it’s very dry.’ P9*

## Discussion

District nurses described challenges around aspects of delirium identification and management when caring for people with terminal illness. Assessment challenges included fluctuating delirium symptoms, complex needs due to patients being referred with more advanced disease than in the past, having limited background information about patients, including problems accessing contemporaneous information, especially out-of-hours. They valued clinical judgement alone when detecting delirium, over formal assessment tools, and rarely used the term ‘delirium.’ Family carers were viewed as essential in caring for the patient, but were often exhausted, and stressed, and needed advice and support themselves. Management challenges related to uncertainty about the underlying causes, delirium associated with opioid toxicity, determining reversibility of the delirium, and palliation of distressing hyperactive symptoms towards the end of life. District nurses identified delirium detection and the pharmacological management of distressing symptoms as priorities for future education and training.

District nurses may be the primary healthcare professionals with most contact with terminally ill people in their homes. As such, they are in a unique position to detect acute changes, including delirium, early, which may improve patient outcomes. However, some participants described a reluctance to use the term ‘delirium’ or uncertainty about the role of delirium detection tools. Similar findings have been described in earlier studies focusing on the practice of specialist palliative care nurses, in the community and in a hospice setting [30, 42]. The UK survey of palliative care doctors in 2019 also reported that detection tools were not in routine clinical practice, with many preferring to use clinical judgement alone [14]. Yet clinical judgement alone is known to under-diagnose delirium, particularly the hypoactive subtype, due to overlapping symptoms with fatigue, depression, and dementia [43–45].

Family carers were recognized as important in identifying delirium and caring for terminally ill people with delirium at home. Carer fatigue and stress were commonly described. Similarly, an integrative literature review described family carers as both providing support to their terminally ill person with delirium, but also requiring support and information themselves [35]. Carers needed information and advice about how best to support the terminally ill person with delirium, as well as when to seek advice from healthcare professionals.

District nurses were familiar with non-pharmacological strategies *to treat* delirium and involved family carers in their implementation with patients. Non-pharmacological strategies in delirium *prevention* were occasionally described. Given the growing evidence of these strategies reducing the onset or severity of delirium [46], family members may benefit from more proactive advice about non-pharmacological prevention. Vardy et al.’s community-based pilot delirium implementation study, which incorporated the delirium TIME management bundle (Triggers, Investigate, Management and Engage) [47], demonstrated that delirium could be safely managed at home, and promoted an increased focus on delirium prevention strategies. Adapting such interventions for terminally ill people may be of value in the future.

We found uncertainty regarding pharmacological palliation of distressing hyperactive symptoms, developing towards the end of life. Use of benzodiazepines and antipsychotics in delirium of mild to moderate severity risks worsening delirium symptoms or adverse effects [18]. However, there is limited research to support management of delirium, particularly towards the end of life. Consequently, guidance recommends medication is reserved for palliation of severe distressing symptoms, unresponsive to other strategies. The uncertainties and complexities of managing distressing symptoms have recently been described by Agar and Amgarth-Duff [3]. Given these challenges, senior clinician support has been recommended in pharmacological management; yet district nurses in this study reported difficulties, at times, accessing advice and support from other primary health care professionals and specialist palliative care services.

District nurses identified educational and training needs, specifically in delirium detection, investigation of reversible underlying causes, as well as management of distressing symptoms towards the end of life. Our findings suggest that education delivered remotely to small groups would be acceptable to district nurses. Indeed, there is growing evidence for the use and value of digital interventions as a mode of delivery for education and training [48]. Models such as Project ECHO, which uses videoconferencing technology to support and train healthcare professionals remotely, may be of value. Project ECHO is an effective and accessible distance learning model, which enables primary care healthcare professionals to acquire new knowledge and skills within palliative care [49, 50]. Such an approach may be of value in training and upskilling district nurses on delirium assessment and management for patients approaching the end of life.

The district nurses in this study clearly identified stress, exhaustion, and distress for patients and their families, when delirium at the end of life causes agitation, which is difficult to manage. In the context of existing pressures on health and social care provision, it is crucial to identify when lack of access to responsive social care and specialist support contributes to poor outcomes, such as unplanned admissions and/or the family and professionals’ perceptions of a ‘bad death.’ Making delirium identification and management a priority for already overstretched services, requires evidence of the financial and psychosocial impact of inadequate management of this common issue, for terminally ill people wishing to spend their final days at home or in a care home setting.

### Strengths and limitations of the study

Participants were recruited from both in-hours and out-of-hours settings, from rural and urban areas across Scotland, thus providing a range of perspectives. Most had been in practice for more than 8 years, and so the study likely reflects the perspectives of experienced nurses, who were already interested and had experience of managing patients with delirium. Inclusion of less experienced district nurses might have revealed lower levels of awareness of what delirium is and how to manage it, as well as different support needs. The term ‘terminally ill’ may have been perceived as alluding to the end-of-life phase (last days of life), which may have limited the breadth of discussion by district nurses. The interviewer was aware of this, and did seek to clarify and broaden the scope of discussion to involve those patients with longer disease prognoses.

### Future research

Future research involving less experienced nurses and those from more culturally and ethnically diverse backgrounds would inform a more heterogeneous range of perspectives. Most delirium research focuses on inpatient settings – consequently future research should include community-based palliative care. The development, evaluation, and implementation of multicomponent delirium management interventions for community-based patients and families are needed. Educational interventions, that can be delivered online and at relatively low cost, have much potential [50] and require evaluation. Further studies are required to implement routine delirium assessment in community settings for people approaching the end of life.

### Implications for practice

Easily accessible education and training of district nurses may be beneficial to further develop expertise in delirium practice. A pragmatic approach to delirium screening amongst terminally ill patients may be to advocate detection tool use at times of elevated risk of developing delirium (for example, when there is a clinical change in the patient’s condition, change in medication or change in care setting). Increased awareness of the hypoactive subtype amongst district nurses, which is associated with poorer outcomes in terminally ill populations, may be beneficial in detecting delirium at an earlier stage, thus improving patient outcomes. Improved clarity of delirium guidance is required, in terms of terminology used and management. Organizational changes are required, involving the resourcing and structure of social care, primary and specialist palliative care services, to provide more responsive support to district nurses, caring for patients with delirium in the community. Worsening financial pressures, in the context of predicted increased community palliative care need, make this evidence critical to appraise and act on.

## Conclusions

District nurses described challenges around aspects of delirium identification and management when caring for people with terminal illness. Online educational interventions and more timely support from specialist palliative care services may develop district nurses’ confidence in supporting terminally ill people, who are at risk of developing, or are experiencing delirium. Multicomponent interventions, focused on delirium assessment and management in the community, need developed, evaluated, and implemented. This will support high quality palliative care for terminally ill people, and their family carers, at home.

## Electronic Supplementary Material

Below is the link to the electronic supplementary material.

Supplementary Material 1

## Data Availability

Interview transcripts can be made available on reasonable request from the author EA.
