# Supplementary material for: What are the experiences and support needs of district nurses caring for terminally ill people with delirium at home? A qualitative study
Source: BMC Palliat Care. 2025 Mar 8;24:60. doi: 10.1186/s12904-024-01627-9 (PMC11889912; doi:10.1186/s12904-024-01627-9)
Supplement: Supplementary file 1 — Supplementary Material 1 [file 12904_2024_1627_MOESM1_ESM.docx]

**Table 1: Interview schedule**

| 1. **What is your experience of caring for terminally ill people in the community?** 2. How frequently do you care for terminally ill patients? 3. **What do you understand by the term “delirium” in the context of your terminally ill patients?** 4. **What would make you think a patient had developed delirium?**   a. How would you identify if a patient had delirium?  b. Are you familiar with any tools that are used to screen for or diagnose delirium?   1. Have you ever used them? 2. What’s your experience of these tools? 3. **Are you aware of any advice or strategies for terminally ill patients or their carers to try to prevent delirium?** 4. **Have you experience of caring for terminally ill people with delirium in the community?** 5. Please can you consider a recent example, that you’d like to share? 6. How was their delirium was managed? 7. How would you rate how well their delirium was managed? 8. **When considering terminally ill patients, what strategies are available to manage their delirium. What would prompt you to use these measures?** 9. What non-pharmacological strategies might you consider when caring for patients with delirium? 10. What advice or support would you give to the family carers of a terminally ill patient with delirium? 11. What would prompt you to administer medication to a patient with delirium. 12. Which medications? – If not brought up, mention haloperidol, levomepromazine, midazolam, lorazepam, other etc. 13. **What are the challenges or difficulties in supporting terminally ill patients with delirium at home?** 14. **What would be helpful, if anything, to support district nurses in caring for patients with delirium?** 15. What is the role of specialist palliative care or hospice services in supporting district nurses in caring for patients with delirium. 16. What might help support your patient with delirium to remain at home, if that is their preference? 17. In your experience, what are the factors which most often trigger a hospital admission for a terminally ill patient with delirium? |
| --- |

**Table 2: Participant characteristics**

| Job title | Duration with district nurse services | Pattern of work | | | Non-medical Prescriber (Yes/No) | Gender | Age | Ethnicity | Interview type (individual or in pairs) |
| --- | --- | --- | --- | --- | --- | --- | --- | --- | --- |
|  |  | Daytime | Evening | Overnight |  |  |  |  |  |
| Community staff nurse | Less than 1 year | Yes | Yes | No | No | Female | 45-54 | White | Individual |
| Community staff nurse | 5 – 1 0 years | No | No | Yes | No | Female | 45-54 | White | Individual |
| Community staff nurse | Over 10 years | Yes | No | No | No | Female | 55-64 | White | Individual |
| District nurse | 5 – 1 0 years | Yes | Yes | No | No | Female | 25-34 | White | Pair |
| District nurse | 5 – 1 0 years | Yes | No | No | Yes | Female | 45-54 | White | Individual |
| District nurse | 5 – 1 0 years | Yes | Yes | No | No | Female | 45-54 | White | Pair |
| District nurse | Over 10 years | Yes | No | No | No | Female | 45-54 | White | Pair |
| District nurse | Over 10 years | Yes | No | No | No | Female | 55-64 | white | Pair |
| District nurse | Over 10 years | Yes | No | No | Yes | Female | 45-54 | White | Individual |
| District nurse | Over 10 years | Yes | No | No | Yes | Female | 55-64 | White | Individual |
| District nurse | Over 10 years | Yes | No | No | Yes | Not specified | Not specified | Not specified | Individual |
| District nurse | Over 10 years | No | No | Yes | No | Female | 55-64 | White | Individual |

**Table 3: Themes, categories and codes**

| Themes | Categories | Codes |
| --- | --- | --- |
| **Delirium detection in the community** | Valuing of clinical judgement | Clinical judgement comprehensive  Assessment tools not routinely used  Assessment tools not valued  Little value when patients cant communicate  Tools add little to clinical judgement in terminal phase |
|  | Lack of confidence in using delirium terminology | Delirium ‘label’ not used  Focus on symptoms of delirium (eg distress, confusion) over delirium overall  Agitation more often used than delirium  Fear of mistake/misdiagnosis |
| **Challenges managing delirium in the community** | Limited information | Referred to service late  Referred with advanced disease  Less information out-of-hours |
|  | Complex symptom management as disease progresses | Multiple causes  Question over reversibility  Late referral meant more complex  Agitation common  Pharmacological management  Uncertainty relating to opioid toxicity |
|  | Use of non-pharmacological strategies | Familiarity with non-pharmacological strategies  Environmental strategies  Reassurance for patient and families |
| **Family carers as providers and recipients of support** | Family as care providers | Support assessment  Implement non-pharmacological strategies  Care Co-ordinator  Conflict |
|  | Family as recipient of support | Emotional distress and stress common  Difficult to cope  Exhaustion  Provide information, advice and reassurance |
| **Education, training and support** | Support from health and social care services | Social care support  Primary care support  Specialist palliative care support |
|  | Priorities | Prevention  Earlier identification  Pharmacological management  Reversibility  Persistent symptoms |
|  | Delivery Format | Small groups  Interactive  Online |
